# Molecular Determinants of β-Lactam Resistance in Methicillin-Resistant *Staphylococcus aureus* (MRSA): An Updated Review

**DOI:** 10.3390/antibiotics12091362

**Published:** 2023-08-24

**Authors:** Harshad Lade, Jae-Seok Kim

**Affiliations:** Department of Laboratory Medicine, Hallym University College of Medicine, Kangdong Sacred Heart Hospital, Seoul 05355, Republic of Korea; harshadlade@hallym.ac.kr or harshadlade@gmail.com

**Keywords:** *Staphylococcus aureus*, MRSA, β-lactams, *mecA*, PBP2a, genetic factors

## Abstract

The development of antibiotic resistance in *Staphylococcus aureus*, particularly in methicillin-resistant *S. aureus* (MRSA), has become a significant health concern worldwide. The acquired *mecA* gene encodes penicillin-binding protein 2a (PBP2a), which takes over the activities of endogenous PBPs and, due to its low affinity for β-lactam antibiotics, is the main determinant of MRSA. In addition to PBP2a, other genetic factors that regulate cell wall synthesis, cell signaling pathways, and metabolism are required to develop high-level β-lactam resistance in MRSA. Although several genetic factors that modulate β-lactam resistance have been identified, it remains unclear how they alter PBP2a expression and affect antibiotic resistance. This review describes the molecular determinants of β-lactam resistance in MRSA, with a focus on recent developments in our understanding of the role of *mecA*-encoded PBP2a and on other genetic factors that modulate the level of β-lactam resistance. Understanding the molecular determinants of β-lactam resistance can aid in developing novel strategies to combat MRSA.

## 1. Introduction

**Figure 1:**
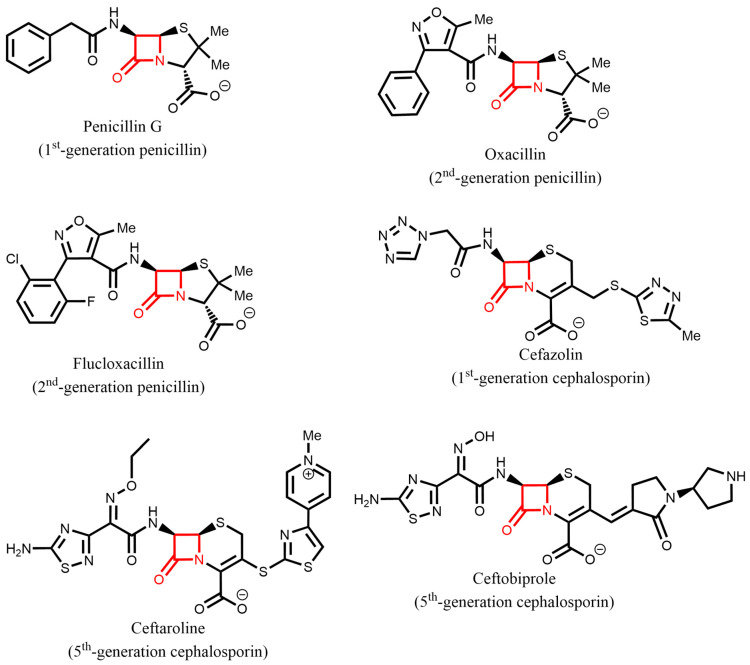
Structures of β-lactams used for the treatment of *S. aureus* infections [4,5]. The core 4-member β-lactam ring is highlighted in red.

*Staphylococcus aureus* is one of the most frequent causes of hospital-acquired and community-acquired infections. *S. aureus* causes a spectrum of diseases from simple skin and soft tissue infections (SSTIs) to more serious conditions such as endocarditis, osteomyelitis, bacteremia, meningitis, and pneumonia [1]. The discovery of β-lactam antibiotic penicillin [2] in 1928, and its subsequent clinical use, which started in 1943, greatly improved the prognosis for patients with staphylococcal infections. Since then, β-lactam antibiotics have remained the gold standard for the treatment of *S. aureus* infections. For many years, β-lactams such as cephalexin and amoxicillin/clavulanate were considered the drug of choice for the treatment of *S. aureus* SSTIs due to their excellent antistaphylococci activity, oral availability, safety, and affordability [3]. However, the propensity of *S. aureus* to acquire resistance to penicillin G has complicated its treatment with β-lactam antibiotics, which now necessitates the use of new-generation cephalosporins such as ceftobiprole and ceftaroline (Figure 1) [4].

The first penicillin-resistant *S. aureus* infection appeared in 1942, initially in hospitals and later in the community [6]. A staphylococcal penicillinase/β-lactamase enzyme that inactivates penicillin was reported in 1944 [7]. The *blaZ* gene encodes a β-lactamase (BlaZ) that hydrolyzes the amide bond of the penicillin β-lactam ring to produce an inactive β-amino acid metabolite [8]. To address the increased prevalence of penicillin resistance in *S. aureus*, methicillin (celbenin), a β-lactamase-insensitive semisynthetic β-lactam antibiotic, was developed and introduced into clinical practice in the United Kingdom in 1959 [9]. However, the first clinical methicillin-resistant *S. aureus* (MRSA) strain appeared as soon as 1960, in a patient with osteomyelitis and septic arthritis in the United Kingdom [10]. Indeed, a recent study using whole-genome sequencing (WGS) of early MRSA isolates (*n* = 209) derived from Public Health England suggested that MRSA may have emerged as early as the mid-1940s [11].

**Figure 2:**
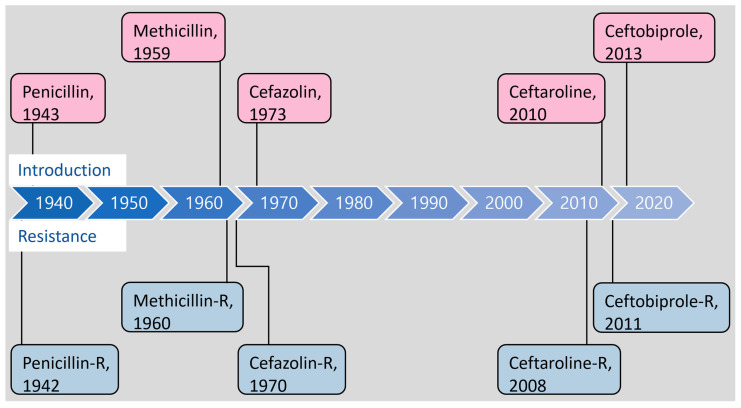
Timeline outlining the introduction of β-lactam antibiotics and the emergence of resistance in *S. aureus*.

Methicillin resistance in *S. aureus* is primarily mediated through the expression of penicillin-binding protein 2a (PBP2a) [12,13], which is encoded by the acquired *mecA* gene located on the Staphylococcal Cassette Chromosome *mec* (SCC*mec*) [14]. PBP2a has a low affinity for most β-lactams except for new-generation cephalosporins and, consequently, performs essential cross-linking of peptidoglycan strands in the presence of β-lactams [12]. Cephalosporins with high affinity for PBP2a were developed to combat MRSA, but resistant strains emerged soon or even before their introduction (Figure 2). MRSA strains are also notorious for their ability to acquire resistance to antibiotics of various other classes, including macrolides, aminoglycosides, tetracyclines, and fluoroquinolones [15,16].

Since its emergence in the 1960s, MRSA has disseminated worldwide and has become a problem in all healthcare settings [17,18]. MRSA is a serious and enduring threat to human health, causing a high rate of morbidity and mortality due to its resistance to antimicrobial treatment [19,20]. In the European Union/European Economic Area (EU/EEA), there were an estimated 170,713 MRSA infections with 6889 attributable deaths in 2016 and 159,670 infections with 6463 attributable deaths reported in 2020 [21]. In 2017, an estimated 323,700 cases of incident hospitalized positive clinical cultures of MRSA, which resulted in 10,600 deaths, were reported in the United States [22]. A cohort study of 890 hospitals in the United States revealed that hospital-onset and community-onset MRSA infections accounted for 52% of all infections caused by multidrug-resistant (MDR) bacteria in 2017 [23]. Moreover, the COVID-19 pandemic has affected antimicrobial resistance in the United States, with available data showing 13% higher hospital-associated MRSA (HA-MRSA) infections in 2020 than in 2019 [24]. In 2019, MRSA caused more than 100,000 deaths and 3.5 million disability-adjusted life-years (DALYs) attributable to antimicrobial resistance [25].

MRSA strains show a unique pattern of β-lactam resistance called heterogeneous resistance [26]. The majority of cells within a bacterial population show resistance to low concentrations of methicillin (≤5 μg/mL), while a small subset of heterogeneous subpopulations exhibits higher methicillin resistance (≥50 μg/mL) [26]. The exposure of such a heterogeneous population of MRSA cells to β-lactam antibiotics selects for cells with higher methicillin resistance, eventually resulting in a homogeneous MRSA population that is entirely resistant to high concentrations of β-lactams [27]. This “hetero-to-homo conversion” of β-lactam resistance is due to increased expression of *mecA* and spontaneous mutations on the chromosome but is not linked to mutations in *mecA* [28,29,30].

## 2. SCC*mec* as a Carrier of Methicillin Resistance

Staphylococcal Cassette Chromosome *mec* (SCC*mec*) is a mobile genetic element (MGE) that carries a *mec* gene complex, consisting of *mecA* (encoding PBP2a) and its regulatory genes *mecR1* (encoding the signal transducer protein MecR1) and *mecI* (encoding the repressor protein MecI) [31,32]. The acquisition of SCC*mec* was the first genetic event in the development of methicillin resistance in *S. aureus* [10]. SCC*mec* inserts, specifically, at the *3*′ end of the *orfX* gene, which encodes an rRNA methyltransferase [31,33]. In MRSA, *mecA* is always located within the SCC*mec* [12,34], while its rare homologs *mecB* and *mecC* are located in plasmids and chromosomes of staphylococci as well as SCC [17,35]. The *S. aureus* LGA251 SCC*mec* type XI strain carries the *mecC* gene, which encodes PBP2c [35,36]. PBP2c shares only 63% amino acid identity with PBP2a [35].

The origin of *mecA* is still unknown, but it is believed to have been acquired from the *Staphylococcus sciuri* species group, which includes *S. fleurettii, S. lentus*, *S. sciuri, S. stepanovicci*, and *S. vitulinus* [37,38,39], whose ecological niches include soil, skin, and the mucous membranes of wild animals. *S. fleurettii* is an animal commensal bacterium that harbors the ancestral *mecA* gene, suggesting that MRSA probably acquired *mecA* from coagulase-negative staphylococci (CoNS) of animal origin [37,40]. The primary function of the original *mecA* gene was probably related to cell wall synthesis, but its evolution into a resistance determinant appears to have occurred via a stepwise process within the *S. sciuri* species group [41].

The SCC*mec* element is characterized by the presence of direct repeats containing integration site sequence (ISS) recognized by cassette chromosome recombinases (ccr) [31,42]. Based on *mec* complex class (A-E), ccr complex types (ccrA, ccrB, and ccrC), and the presence/absence of regulatory genes and insertion sequences (IS), fourteen SCC*mec* types (I–XIV) structural variants have been reported in *S. aureus* to date [43]. The sizes of *SCCmec* elements range between 20 kb to 60 kb or more. These elements are integrated by ccr at a specific site (attachment site, *attB*) in *orfX*, which is localized close to the origin of replication [31,33]. SCC*mec* types II and III are the largest elements and harbor genes that confer resistance to various classes of antibiotics and are most commonly found in HA-MRSA [17,42]. More generally, type IV and V SCC*mec* cassettes are detected in community-associated MRSA (CA-MRSA) strains, such as USA300 and USA400, but also among some widespread HA-MRSA clones of sequence type (ST)5-MRSA-VI, ST22-MRSA-IV, and ST45-MRSA-IV [44]. Transposon Tn554, which carries genes conferring resistance to macrolides, lincosamides, streptogramin B, and spectinomycin, is present in SCC*mec* type II but not in type IV strains [45]. Thus, SCC*mec* type II strains are resistant to multiple antibiotics, whereas type IV strains are resistant to β-lactams but susceptible to other classes of antibiotics.

In clinical settings, the generation of new MRSA clones by the transfer of *mecA* between *S. aureus* strains is rare. The methicillin-sensitive *S. aureus* (MSSA) strain WKZ-1 and the MRSA strain WKZ-2, which were isolated from the same neonate, are isogenic except for the presence of SCC*mec* type IV [46,47,48]. WKZ-2 is thought to have acquired *mecA* DNA, horizontally, from a CoNS isolate also present in the neonate [46].

## 3. Molecular Mechanisms of β-Lactam Resistance

**Figure 3:**
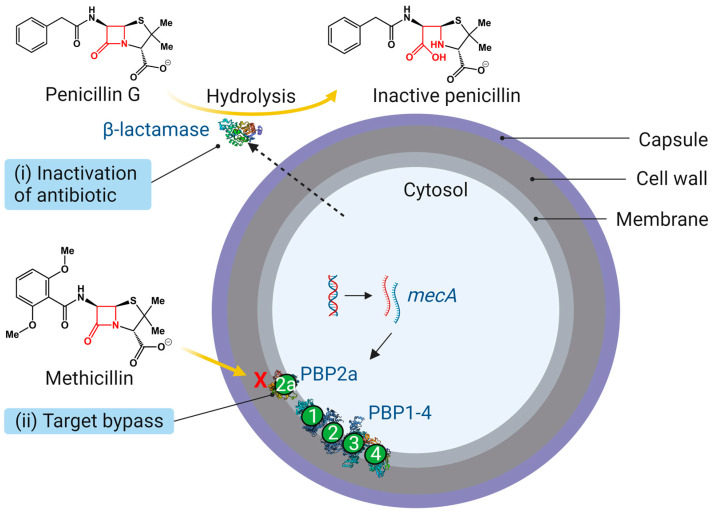
Molecular mechanisms of β-lactam resistance in *S. aureus*. (i) Inactivation of antibiotic: hydrolysis of the amide bond of penicillin G by β-lactamase, rendering the antibiotic inactive [7]. (ii) Target bypass: the TPase activity of PBP2 targeted by β-lactams is taken over by acquired PBP2a in MRSA, which is not inhibited by β-lactams [12].

In *S. aureus*, there are two primary mechanisms of β-lactam resistance: (i) inactivation of antibiotic and (ii) target bypass (Figure 3). In the former, the production of a *blaZ*-encoded β-lactamase (BlaZ) inactivates penicillin G [7], and in the latter, the transpeptidase activity of PBP2 is carried out, predominantly, by MRSA-acquired *mecA*-encoded PBP2a, which has low affinity for β-lactamase-insensitive β-lactam antibiotics such as methicillin and oxacillin [12]. In rare cases, the *mecA* homologs *mecB* and *mecC*, which encode PBP2b and PBP2c, respectively, confer resistance to β-lactams [35,49]. MRSA strains express both β-lactamase and PBP2a [50] and are often resistant to multiple classes of antibiotics.

### 3.1. β-Lactamases

The primary mechanism for β-lactam resistance in *S. aureus* is the production of a β-lactamase (BlaZ), which hydrolyzes the amide bond of the four-membered β-lactam ring in a two-step acylation–deacylation reaction cycle, protecting PBPs from inactivation [7,8]. The *blaZ*-encoded BlaZ is a type A serine β-lactamase [51]. It exhibits high hydrolytic activity against first-generation penicillins but weak activity against second-generation penicillins (e.g., methicillin) and first-generation cephalosporins (e.g., cefazolin) [52].

**Figure 4:**
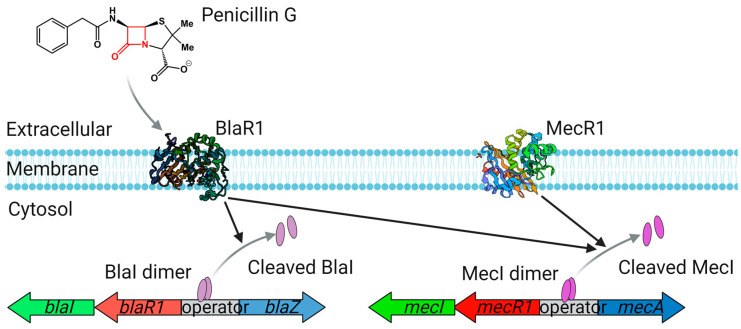
Regulation of *blaZ* and *mecA*. The *bla* or *mec* gene clusters encode regulatory systems that sense β-lactams on the cell surface through a membrane-embedded sensor–inducer BlaR1 (PDB: 1XA7)/MecR1 (PDB:6O9S) [60]. The signal generated by β-lactam detection induces the cleavage of a cytoplasmic transcriptional repressor BlaI/MecI. This results in the expression of *blaZ* and *mecA* genes, which encode BlaZ and PBP2a, respectively.

The 846 bp *blaZ* gene is controlled by two regulatory genes: an antirepressor (*blaR*) and repressor (*blaI*; Figure 4). The genes encoding BlaZ, its repressor BlaI, and a transmembrane sensor-transducer *BlaR1*, are clustered together, either on a plasmid or on the chromosome [53]. Expression of *blaZ* is not constitutive but is induced following exposure of the cell to β-lactam antibiotics [54]. In clinical *S. aureus* strains, the membrane-embedded receptor BlaR1 senses β-lactams through the acylation of its sensor domain [55], inducing transmembrane signaling and activation of a cytoplasmic-facing zinc metalloprotease domain [56]. The activated zinc metalloprotease domain then cleaves the repressor protein BlaI [57,58], inducing the expression of β-lactamase [59,60].

Staphylococcal β-lactamases are categorized into four types (A-D) based on serotyping and substrate specificity [61,62]. Type A serine β-lactamases are the most common in *S. aureus*, while the other types differ according to their ability to hydrolyze different substrates, which include some cephalosporins [63,64]. For example, type A and D β-lactamases are more efficient at hydrolyzing cefazolin and nitrocefin, while types B and C are more efficient against cephalothin [62].

### 3.2. Penicillin-Binding Proteins (PBP1-4 and PBP2a)

Penicillin-binding proteins (PBPs) are a family of membrane-bound proteins involved in the final steps of bacterial cell wall assembly [65,66]. They are essential for the synthesis of peptidoglycan, the main component of the cell wall. PBPs catalyze the polymerization of glycan chains and cross-link them into a mesh-like hydrogel through their transglycosylase (TGase) and transpeptidase (TPase) activities, respectively [67]. Penicillin and other β-lactam antibiotics bind to PBPs, especially via the TPase domain, and prevent them from cross-linking peptidoglycan chains, leading to a weakened cell wall and subsequent bacterial death.

**Figure 5:**
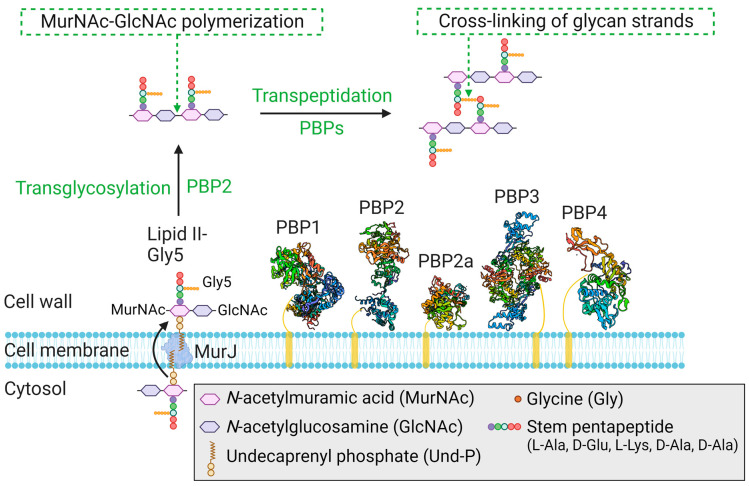
Structural and organizational representation of PBPs that play a crucial role in the final stages of peptidoglycan synthesis in *S. aureus*. The crystal structures of PBP1 (5TRO), PBP2 (2OLU), PBP2a (1VQQ), PBP3 (3VSK), and PBP4 (6C39) are shown. Endogenous PBP1-4 and acquired PBP2a all have a TPase domain, while PBP2 is unique in also possessing a TGase domain. After flipping to the outer side of the cytoplasmic membrane by MurJ, the peptidoglycan precursor Lipid II-Gly5 can be polymerized by the TGase activity of PBP2, and the glycan strands are then cross-linked by the TPase activity of the PBPs [65].

*S*. *aureus* normally possesses four endogenous PBPs, PBP1, PBP2, PBP3, and PBP4 [68], with an additional, acquired PBP2a found in MRSA (Table 1) [12]. All four endogenous PBPs are known to localize at the cytoplasmic membrane, the site of peptidoglycan synthesis [69,70,71]. Each endogenous PBP possesses TPase activity [66], whereas PBP2 alone has an additional, distinct catalytic domain for TGase activity [72]. The TGase activity of PBP2 promotes the polymerization of Lipid II-Gly5 and, subsequently, the TPase activity of PBPs cross-links glycan strands via flexible peptides (Figure 5) [65,72]. Monofunctional glycosyltransferases SgtA and SgtB also have TGase activity, but only SgtB can support *S. aureus* growth in the absence of the main TGase activity of PBP2. However, SgtB cannot support bacterial growth in the presence of β-lactams, in which case an interaction between PBP2 and PBP2a is required [73].

In *S. aureus*, PBP1 (TPase activity only) and PBP2 (both TGase and TPase activity) are essential and sufficient for septal and peripheral peptidoglycan synthesis [68]. In septal peptidoglycan synthesis, PBP1 enables the formation of the septal plate, which is essential for septum architecture [74]. PBP2 is the only bifunctional enzyme in *S. aureus* which catalyzes the polymerization of Lipid II-Gly5 and cross-linking of glycan strands [72]. PBP3, which possesses TPase activity only, is nonessential and its loss does not affect either cell growth or survival [81]. PBP3, which has a C-terminal penicillin-binding domain and a well-conserved N-terminal domain, is more sensitive to methicillin than either PBP1 or PBP2 [81]. PBP3 participates with the shape, elongation, division, and sporulation (SEDS) family of proteins to maintain cell shape (e.g., RodA-PBP3) [79]. PBP4 possesses TPase activity only and is required for the synthesis of highly cross-linked peptidoglycan [70].

PBP2a has low affinity for β-lactams and effectively compensates for the inhibition of TPase activity of PBP2 by β-lactam antibiotics [77,82], allowing it to perform essential cross-linking of peptidoglycan chains. This cooperation between PBP2 and PBP2a allows MRSA to survive, even in the presence of β-lactams. PBP2a is composed of an N-terminal TPase domain and a C-terminal PBP dimer [83]. PBP2a expression depends on the presence of functional MecI/MecR1 regulators in the *mec* operon (Figure 4), but the level of β-lactam resistance does not always correlate with PBP2a expression [84,85]. The expression of PBP2a does not affect the levels of other PBPs in the MRSA strain RN450M (ATCC 8325-1 transformed with the COL *mec* region; Bla^–^) [86] and PBP2a cannot replace the essential function of PBP1 in the MRSA strain RN4220 COL in vitro [69].

### 3.3. PBP2a Mutation and New-Generation Cephalosporin Resistance

The rapid development of resistance to new-generation cephalosporins has not been observed in either clinical MRSA or in in vitro studies, though some rare ceftaroline-resistant (EUCAST MIC ≥ 1 μg/mL or CLSI MIC ≥ 4 μg/mL) clinical MRSA isolates have been reported [87,88,89]. Ceftaroline resistance was found in 40 out of 60 archived HA-MRSA ST228 (South German clonotype) and ST247 (Iberian clonotype) clinical strains isolated in western Switzerland since 1998 [88]. Missense mutations in the PBP2a allosteric domain (N146K or E239K and N146K-E150K-G246E) are responsible for the ceftaroline susceptibility of these isolates [88] (Table 2). High-level ceftaroline-resistant (MIC ≥ 32 μg/mL) MRSA ST5 clinical isolates from the United States harbor two amino acid-altering mutations (Y446N and E447K) in the ceftaroline-binding pocket of the TPase domain of PBP2a [90]. Other ceftaroline-resistant (MIC 8 μg/mL) MRSA isolates (*n* = 12) predominantly belong to SCC*mec* type IV and contain a substitution (E447K) in the TPase domain of PBP2a [91]. High-level ceftaroline-resistant (MIC ≥ 16 μg/mL) MRSA ST5 with *spa* type t111 blood isolates (*n* = 10) collected from eight hospitals in South Korea, in 2017, revealed five amino acid substitutions in PBP2a, including three (E447K, I563T, and S649A) in the TPase domain and two (N104K and V117I) in the non-penicillin binding domain [92]. The accumulation of substitutions in PBP2a was found to be associated with ceftaroline resistance in these isolates.

Laboratory MRSA strains with reduced susceptibility to cephalosporins following exposure to increasing concentrations of antibiotics were analyzed to identify the mutations responsible for β-lactam resistance. The ceftaroline-passaged SF8300 MRSA mutant carries a single *mecA* mutation E447K and expresses low-level ceftaroline resistance, while the COL ceftaroline-passaged mutant exhibits high-level resistance to both ceftobiprole and ceftaroline and has mutations in *pbp2*, *pbp4*, and *gdpP* but not in *mecA* [93].

**Table 2:** Molecular determinants of β-lactam resistance in MRSA.

β-Lactam Antibiotics	Year of Discovery/ Introduced or Approved	Year of Resistance Reported	Resistance Determinant/Gene Product
Penicillin G	1928 [2]/1943	1942 [6]	*blaZ*/BlaZ [7] (β-lactamase hydrolyzes the peptide bond in the β-lactam ring)
Methicillin ^#^	1959 [9]/--	1960 [10]	*mecA*/PBP2a [12,13] (PBP2a has a low affinity for methicillin)
Oxacillin	1960/U.S. FDA 1971		*mecA*/PBP2a
Cefoxitin *	1974 [94]/U.S. FDA 1978		*mecA*/PBP2a
Cefazolin	1970 [95]/U.S. FDA 1973	1970 [96]	*mecA*/PBP2a [97]
Ceftaroline	2003 (Ceftaroline fosamil) [98]/U.S. FDA 2010, E.U. EMA 2012	2013 [90]	*mecA*/PBP2a Mutations in *mecA* [88,90], *pbp2*, *pbp4* [93,99,100], *clpX,* and *gdpP* [93,101]
Ceftobiprole	1998 [102]/E.U. EMA 2013	2008 [103]	*mecA*/PBP2a Mutations in *mecA* [103], *pbp2*, *pbp4* [93,104], *clpX*, and *gdpP* [93,101]

^#^ Methicillin is no longer used for MRSA therapy due to its toxicity; * cefoxitin is used for screening only.

## 4. Genetic Factors Modulating β-Lactam Resistance

Upon acquisition of *mecA* into the *S. aureus* chromosome, PBP2a can no longer function independently of the endogenous processes that regulate cell homeostasis [105] and requires the integration of multiple genetic factors [106,107]. The genes/operons regulating cell wall synthesis, cell division, cell signaling, and metabolism alter the level of PBP2a expression in MRSA [108,109]. Induced mutation studies have revealed that mutations in genes/operons related to protein stability (*clpXP*), nucleotide signaling (*gdpP*), RNA polymerase activity (*rpoB/rpoC*), quorum sensing (*agr*), cell signaling (*sarA*), cell division (*stk1/stp1* and *ftsH*), precursors for cell wall synthesis (*auxA* and *auxB*), peptidoglycan biosynthesis (*pbp4*), cell division (*ftsZ)*, and cell wall homeostasis (*dltA*) all alter the level of β-lactam resistance in MRSA [110,111]. Furthermore, transposon mutagenesis studies have identified several genes that are unlinked to *mecA* but whose function is essential for methicillin resistance [106,112,113].

### 4.1. ClpXP System

The highly conserved ClpX chaperone is an ATP-dependent unfoldase that interacts with ClpP proteolytic subunits to form the ClpXP complex [114]. ClpX targets proteins within cells for degradation by associating with ClpP peptidase. Furthermore, ClpX subunit can also function independently by facilitating the correct folding of newly synthesized proteins and maintaining their structure [114]. The deletion of *clpX* in *S. aureus* inhibits its growth by inhibiting specific steps in the biosynthesis pathways of peptidoglycan and teichoic acids [115,116]. In *S. aureus*, the proteolytic activity of ClpXP is required for the production of virulence factor Protein A [117] and modulation of β-lactam resistance [118,119]. The inactivation of *clpX* or *clpP* increases the β-lactam resistance of the *S. aureus* USA300 strain and simultaneously affects cell envelope properties [118]. Moreover, mutations in ClpX, induced by passaging *S. aureus* in the presence of β-lactam antibiotics, contribute to high-level resistance to ceftaroline and ceftobiprole [101].

### 4.2. Cyclic-di-AMP Phosphodiesterase (GdpP)

GdpP is a phosphodiesterase enzyme that cleaves the second messenger cyclic-di-AMP [120]. It regulates bacterial cell size to help cope with extreme membrane stress and cell wall stress [121]. Loss-of-function mutations in *gdpP* lead to decreased susceptibility of *S. aureus* to β-lactams [122]. Methicillin-resistant isolates lacking the *mecA* gene have been reported since the 1980s [123,124]. These phenotypes result from the hyperproduction of β-lactamase, which partially hydrolyzes β-lactamase-resistant penicillin [125]. The loss of *gdpP* gene function leads to β-lactam tolerance and enhanced evolution of β-lactam resistance in *S. aureus* [126]. A laboratory-generated *mecA*-negative strain, CRB, obtained after high inoculum serial passage of the COL strain in ceftobiprole, exhibited resistance to many β-lactams but was hypersusceptible to cefoxitin [103,124] and showed five single-nucleotide polymorphisms (SNPs) in three genes: *pbp4*, *acrB*, and *gdpP*.

### 4.3. RNA Polymerase (RpoB/RpoC)

The *rpoB* and *rpoC* genes encode the largest subunits of RNA polymerase β and β′, respectively. The role of *rpo* mutations in the conversion from heterogeneous to homogeneous methicillin resistance has been reported [127]. Mutations in *rpoB* and *rpoC* genes result in the upregulation of eleven genes, including *mecA*, and the downregulation of genes associated with anaerobic and fermentative respiration, resulting in high-level β-lactam resistance in MRSA [127]. However, no direct correlation between PBP2a levels and antibiotic resistance has been reported. An *rpoB* mutation (N967I) in MRSA strain N315 and its derived strains, following passage with imipenem, resulted in a heterogeneous-to-homogeneous phenotypic conversion of β-lactam resistance (oxacillin MIC 4 to ≥256 μg/mL) [128].

### 4.4. Accessory Gene Regulator (Agr) System

In *S. aureus*, the global regulator *agr* primarily controls biofilm formation and the production of toxins, such as the phenol-soluble modulins [129], and is reported to modulate methicillin resistance in *S. aureus* [130]. An *S. aureus* USA300-0114 (LAC) *agr* mutant strain showed higher resistance to oxacillin and ampicillin, associated with the constant expression of *mecA*, increased concentrations of long-chain fatty acids in the cytoplasmic membrane, and the thickening of biofilms [131], while inactivation of either *agr* and/or *sar* in heterogeneously methicillin-resistant MRSA resulted in a small but reproducible decrease in the number of cells in the subpopulation expressing high methicillin resistance [130]. In heterogeneous clinical MRSA strains, a temporally controlled increase in *agr* expression is required to tightly modulate SOS-mediated mutation rates, resulting in the full expression of oxacillin homogeneous resistance [132]. However, this two-component regulatory system (TCS) has a complex network, and the exact mechanism remains unclear.

### 4.5. Staphylococcal Accessory Regulator A (SarA)

SarA is a 14.7 KDa DNA-binding protein (124 residues) encoded by the *sarA* locus, containing *sarAP2*, *sarAP3*, and *sarAP1* that regulates the transcription of a variety of virulence genes by binding to the promoter region of its target genes [133,134]. The global regulator SarA promotes the synthesis of specific extracellular and cell wall-associated proteins [135,136]. SarA has been reported to regulate β-lactam resistance in MRSA in vitro and in endovascular infections [137]. The inactivation of *sarA* in *S. aureus* JE2 decreased *mecA* expression, resulting in a significant reduction in oxacillin resistance [137,138]. A recent study demonstrated that SarA positively controls *mecA* expression by binding to the *mecA* promoter [138,139], indicating this TCS may directly regulate β-lactam resistance.

### 4.6. Serine/Threonine Kinase and Phosphatase (Stk1/Stp1)

The coordinated phosphorylation/dephosphorylation reactions carried out by the *stk1*-encoded eukaryotic-like serine/threonine kinase (Stk1) and the *stp1*-encoded cognate phosphatase (Stp1) play a crucial role in cell division and morphogenesis [140,141,142]. Stk1 is a membrane-bound protein and Stp1 is a cytosolic protein. Both proteins generally function together to regulate the reversible phosphorylation of substrates [143,144]. The kinase-phosphatase pair Stk1-Stp1 mediates the phosphorylation of reactive cysteine residues, which is crucial in regulating virulence determinants and resistance to cell wall-targeting antibiotics [145]. An *S. aureus* N315 mutant lacking both Stk1 and Stp1 exhibited increased sensitivity to cephalosporins (e.g., cefazolin, cefotaxime, ceftriaxone) due to significant cell division defects such as multiple and incomplete septa, bulging, and irregular cell size [141], while loss-of-function point mutations in the *stp1* gene facilitated β-lactam resistance in laboratory-passaged *S. aureus* isolates that lacked both *mecA* and *blaZ* [146].

### 4.7. FtsH Protease

FtsH is a membrane-bound ATP-dependent zinc metalloprotease that plays a role in resistance to stresses such as nutrient starvation, acidity, and toxic chemicals [147]. FtsH sensitizes MRSA to β-lactams by degrading YpfP, an enzyme responsible for the synthesis of an anchor molecule for lipoteichoic acid (LTA) [148]. The *ftsH* gene is involved in the regulation of cell division and its deletion increases the production of normal LTA, making MRSA more resistant to β-lactams [148].

### 4.8. AuxA and AuxB

The auxiliary (*aux*) genes or factors assist PBP2a in conferring β-lactam resistance but are not the sole mediators of resistance. The Aux group of components provides precursors necessary for correct cell wall synthesis and also includes factors involved in various cellular physiological processes, including nitrogen metabolism (GlnR repressor) [149], fatty acid biosynthesis (acyl carrier protein HmrB) [150], and lysinylation of phosphatidyl glycerol in the cell membrane (FmtB and FmtC/MprF) [151,152]. AuxA and AuxB mutant MRSA strains show increased susceptibility to β-lactams, but no changes in PBP2a expression, peptidoglycan cross-linking, or wall teichoic acid synthesis [153].

### 4.9. PrsA

PrsA is a membrane-bound post-translocational chaperone lipoprotein involved in the folding and secretion of many cell surface-associated proteins, including PBP2a in MRSA [154]. Deletion of *prsA* decreases oxacillin resistance in different SCC*mec* type strains (COL, Newman, Mu3) and causes a decrease in PBP2a membrane levels, without affecting *mecA* mRNA levels [154]. PrsA, together with another membrane-bound chaperone/serine protease, HtrA1, plays an important role in the post-transcriptional maturation of PBP2a [155], which is probably related to the export and/or folding of newly synthesized PBP2a. Dual disruption of PrsA and HtrA1 in *S. aureus* strain COL resulted in synergistic attenuation of PBP2a folding that restored oxacillin sensitivity [155]. A spot population analysis profile assay revealed that the *prsA*-deleted MRSA strain COL exhibited a significant decrease in oxacillin resistance [156].

### 4.10. PBP4

PBP4 acts to remodel peptidoglycan but is not essential for *S. aureus* growth [52]. A *pbp4* knockout resulted in reduced cross-linked muropeptide in *S. aureus* [157]. The loss of PBP4 alone caused a sixteen-fold decrease in oxacillin and nafcillin resistance in two common CA-MRSA isolates, USA300 and MW2, indicating that PBP2a is not the sole factor for methicillin resistance in CA-MRSA [158]. The loss of PBP4 also decreased the levels of PBP2 in these CA-MRSA strains after a challenge with oxacillin, resulting in a significant reduction in peptidoglycan cross-linking [158]. A laboratory-passaged *S. aureus* COLn strain lacking the *mecA* gene developed high-level resistance to β-lactams, due to *pbp4* promoter mutations that resulted in the overexpression of PBP4 and a highly cross-linked cell wall [159]. The *pbp4* promoter mutation in CRB (COLnex passaged in ceftobiprole) caused increased membrane levels of PBP4 and resulted in a highly cross-linked cell wall [100]. Moreover, the CRB strain developed high-level resistance to new-generation cephalosporins such as ceftobiprole and ceftaroline.

### 4.11. Filamentous Temperature-Sensitive Protein Z (FtsZ)

The cell division protein FtsZ is highly conserved in *S. aureus* and is a major component of the divisome [160]. FtsZ migrates to the division site and self-polymerizes, forming protofilaments that aggregate into a filamentous ring-like structure called the Z-ring. The Z-ring acts as a cytoskeletal scaffold, recruiting and organizing other cell segregation proteins to facilitate septum formation and cell division [161]. The reduced expression of cell division genes (*ftsA*, *ftsW*, and *ftsZ*) sensitizes MRSA to β-lactam antibiotics [161]. FtsZ has been reported to modulate PBP2a expression, thus affecting the resistance of MRSA to β-lactams [162].

### 4.12. D-alanyl Carrier Protein Ligase DltA

The *dltA*-encoded DltA is involved in the addition of D-alanine to TAs. Deletion of the *dltA* gene within the *dltABCD* operon prevents the attachment of d-alanyl esters to both LTA and WTA, resulting in increased sensitivity of the mutant to host defense peptides [163]. An *S. aureus* MW2 *dltA* mutant showed decreased resistance to oxacillin and amoxicillin, suggesting that the loss of D-alanylation of TAs increases MRSA sensitivity to β-lactams [164].

## 5. Conclusions

The resistance of MRSA to β-lactam antibiotics is mainly mediated by acquired *mecA*-encoded PBP2a, which takes over the essential TPase activities of endogenous PBPs when they are inhibited by β-lactams. In addition, other genetic factors, including *ClpXP,* GdpP, RpoB/RpoC, Agr, SarA, Stk1/Stp1, FtsH, AuxA and AuxB, PrsA, FtsZ, PBP4, and DltA, which are involved in protein stability, nucleotide-signaling, genetic information processing, quorum sensing, cell signaling, cell division, protein folding and stabilization, cross-linking of glycan strands, and cell wall homeostasis, also contribute to β-lactam resistance in MRSA. Since changes in several molecular determinants associated with the fundamental physiology of MRSA alter its β-lactam resistance, a greater understanding of the interactions between PBP2a and these genes should help to identify a range of potential targets for the development of new therapies to treat MRSA infections.

## Figures and Tables

**Table 1:** Classification of PBPs involved in the cell wall biosynthesis of *S. aureus*.

Protein(s)	Gene(s)	Class	Activity	Function/Relevant Features
PBP1	*pbp1*	HMM class B	TPase	PBP1 is an essential protein that plays a crucial role in cell division and cell wall integrity [69,74].
PBP2	*pbp2*	HMM class A	TPase and TGase	PBP2 is an essential protein that acts as the major peptidoglycan synthase responsible for cell wall synthesis [75,76].
PBP2a	*mecA*	HMM class B	TPase	PBP2a is an acquired protein [12] that compensates for the loss of endogenous PBP2 TPase activity in the presence of β-lactams [77].
PBP3	*pbp3*	HMM class B	TPase	PBP3 is a nonessential protein for growth [78] and is associated with septal localization of RodA [79]; how PBP3 modulates RodA activity (cell elongation and maintenance) remains unclear.
PBP4	*pbp4*	LMM class C	TPase	PBP4 is a nonessential protein for growth and is involved in the regulation of cross-linking within peptidoglycan [70,80].

HMM: high molecular mass; LMM: low molecular mass; TGase: transglycosylase; TPase: transpeptidase.

## Data Availability

The data presented in this study are available in the article.
